# Development of PEG-Mediated Genetic Transformation for Pine-Inhabiting Fungus *Pestalotiopsis disseminata*

**DOI:** 10.3390/jof12100741

**Published:** 2026-10-03

**Authors:** Zhaojie Zhan, Quanchao Wang, Haonan Wang, Eeva Terhonen, Lifeng Zhou, Oleg A. Kulinich, Huachao Xu, Xudong Zhou

**Affiliations:** 1 State Key Laboratory for Development and Utilization of Forest Food Resources, College of Forestry, Zhejiang A&F University, Hangzhou 311300, China; zhanzj@stu.zafu.edu.cn (Z.Z.); wangquanchao@stu.zafu.edu.cn (Q.W.); haonanwang@zafu.edu.cn (H.W.); lf.zhou@zafu.edu.cn (L.Z.); xhcinsect@stu.zafu.edu.cn (H.X.); 2 Natural Resources Institute Finland (Luke), Forest Health and Biodiversity, Latokartanonkaari 9, 00790 Helsinki, Finland; eeva.terhonen@luke.fi; 3 Department of Forest Quarantine, All-Russian Center of Plant Quarantine, Moscow 140150, Russia; okulinich@mail.ru

**Keywords:** protoplast, PEG-mediated transformation, green fluorescent protein, genetic transformation

## Abstract

*Pestalotiopsis disseminata* widely inhabits pines, but the lack of an efficient genetic transformation system has limited its functional genomic studies. Here, we optimized protoplast preparation and established a PEG-mediated transformation and GFP-labeling platform for strain ZXD579. The selected conditions used mycelia cultured for 24 h and digested for 3 h at 28 °C with 1% lysing enzyme, 0.1% driselase, and 1% lysozyme in 0.6 M KCl supplemented with 50 mM CaCl_2_. The protoplast yield reached (1.51 ± 0.15) × 10^7^ protoplasts/mL, and the regeneration rate exceeded 90%. Hygromycin B at 130 μg/mL completely inhibited wild-type growth and was suitable for transformant selection. The pCT74-eGFP plasmid carrying the reporter gene and hygromycin B phosphotransferase gene (*hph*) was introduced into protoplasts using PEG. Putative transformants were examined by PCR and fluorescence microscopy; GFP fluorescence was detected in hyphae and conidia. Transformation efficiency was 4.2 ± 0.6 transformants per μg plasmid DNA, and 60% of 15 randomly selected hygromycin-resistant transformants were PCR-positive. GFP-labeled transformants showed no significant difference in vegetative growth and exhibited comparable preliminary pathogenicity phenotypes relative to the wild-type strain. This protocol provides a foundation for gene manipulation in *P. disseminata*.

## 1. Introduction

The genus *Pestalotiopsis* (Ascomycota, Xylariales, Amphisphaeriaceae) encompasses a diverse assemblage of fungi that are globally distributed and exhibit multiple ecological lifestyles, such as plant pathogens, endophytes, or saprophytes [1]. Among them, *P. disseminata* has been implicated in severe diseases affecting multiple economically important crops. For instance, it causes grey blight of *Euonymus japonicus* in China, with disease incidence reaching 52–70% [2]; peach gummosis in India, leading to up to 85% fruit loss [3]; guava fruit scab [4]; and grey blight of *Persea bombycina*, the host plant of the muga silkworm [5]. The species is also a well-known endophyte of asymptomatic plants [6,7], and its phytosanitary relevance is further underscored by a recent EFSA pest categorisation [8]. Recently, the fungus was reported as the causal agent of needle blight on *Pinus massoniana* in China [9]. Further, *P. disseminata* also produces structurally diverse secondary metabolites [10], demonstrating insecticidal activity [11]. Despite its ecological and economic importance, molecular studies on *P. disseminata* remain limited because no genetic manipulation platform has been established for this fungus. With the recent availability of genomic resources for *P. disseminata*, including a draft genome sequence [12], the functional characterization of genes related to development, pathogenicity, and secondary metabolism is central to modern mycology [13,14]. Genetic transformation underpins these studies by enabling gene knockout, overexpression, complementation, and protein localization analysis [15,16,17]. Green fluorescent protein (GFP) has been extensively employed as a crucial reporter to monitor protein dynamics and pathogen–host interactions in living cells [18,19].

Among the available fungal transformation methods, polyethylene glycol (PEG)-mediated protoplast transformation is widely used because it is technically straightforward and requires no specialized equipment [15]. The success of this approach depends strongly on the yield and viability of protoplasts, which are influenced by multiple factors including mycelial age, lytic enzyme composition, osmotic stabilizer type, digestion time, and temperature [13,20]. Given that fungal cell wall composition varies significantly among species, no single protocol can be universally applied, and thus species-specific optimization is necessary [21]. The combination of lysing enzyme and driselase has been demonstrated to be effective for protoplast release in several filamentous fungi [22,23], yet it remains unknown whether this or other enzyme formulations are suitable for *P. disseminata*.

Therefore, this study aimed to optimize the key parameters governing protoplast preparation and regeneration, establish a PEG-mediated genetic transformation protocol using the pCT74-eGFP vector, and evaluate the growth, GFP expression, stability during serial subculture, and preliminary pathogenicity phenotypes of GFP-labeled transformants. The resulting platform is intended to support functional gene analysis and live-cell imaging of *P. disseminata* during host infection.

## 2. Materials and Methods

### 2.1. Fungal Strain, Plasmid Vector, and Culture Conditions

The pine needle blight pathogen *P. disseminata* ZXD579 was used throughout this study. The strain was originally isolated from diseased needles of *P. thunbergii* in Shandong Province, China, and identified by the internal transcribed spacer (ITS), translation elongation factor 1-alpha (*tef1-α*), and beta-tubulin (*tub2*) regions [9]. The corresponding GenBank accession numbers are PV259792 (ITS), PV275113 (*tef1-α*), and PV275187 (*tub2*). The strain is maintained in the laboratory collection of the Forest–Insect–Microbe Interaction research team, Zhejiang A&F University. Stock cultures were maintained on malt extract agar (MEA; 20 g malt extract and 20 g agar per liter of water) at −20 °C, and fresh cultures were prepared on MEA at 28 °C before each experiment.

The plasmid pCT74, commercially obtained from Wuhan Miaoling Biotechnology Co., Ltd. (Wuhan, China; Cat. No. P71037), was used as the transformation vector. The vector carries the hygromycin B phosphotransferase gene (*hph*) as a selection marker and the *eGFP* reporter gene driven by the ToxA promoter [24]. The vector backbone is approximately 5790 bp and carries an ampicillin-resistance gene for bacterial selection.

### 2.2. Media, Reagents, and Buffers

The media and buffers used in this study were as follows. For routine cultivation of *P. disseminata*, malt extract agar (MEA) was used. For mycelial production, potato dextrose broth (PDB; 26 g PDB powder in 1 L ddH_2_O) was employed. RM medium (6 g yeast extract, 6 g casamino acids, 200 g sucrose, and 15 g agar for solid medium, per liter of ddH_2_O) was used for protoplast regeneration and transformant selection.

Four osmotic stabilizers were tested: 0.7 M NaCl, 0.6 M KCl, MN buffer (0.3 M MgSO_4_ + 0.3 M NaCl), and 0.6 M KCl supplemented with 50 mM CaCl_2_. STC buffer (1.2 M sorbitol, 50 mM Tris-HCl, pH 8.0, and 50 mM CaCl_2_) was used to wash and resuspend protoplasts. SPTC buffer was prepared by dissolving 40% (*w*/*v*) PEG 4000 in STC buffer; the solution was heated at 65 °C for 20 s and filter-sterilized through a 0.22-μm membrane.

The lytic enzymes were lysing enzyme (from *Trichoderma harzianum*; Sigma-Aldrich, St. Louis, MO, USA; Cat. No. V2010), driselase (Sigma-Aldrich, St. Louis, MO, USA; Cat. No. D8037), lysozyme (Beijing Solarbio Science & Technology Co., Ltd., Beijing, China; Cat. No. 14240321001), and cellulase (Beijing Solarbio Science & Technology Co., Ltd., Beijing, China; Cat. No. C8270). Enzyme working solutions were dissolved in the appropriate osmotic stabilizer immediately before use and filter-sterilized through a 0.22-μm membrane.

### 2.3. Determination of the Minimal Inhibitory Concentration of Hygromycin B

To determine the appropriate selection pressure for hygromycin-resistant transformants, the sensitivity of wild-type *P. disseminata* ZXD579 strain to hygromycin B was evaluated. Mycelial plugs of 5 mm diameter were excised from the actively growing margin of 3-day-old colonies and transferred onto MEA plates supplemented with 0, 10, 20, 30, 50, 70, 110, 130, or 150 μg/mL hygromycin B. Plates were incubated at 28 °C in the dark for 5 days. The minimal inhibitory concentration (MIC) was defined as the lowest concentration that completely inhibited visible mycelial growth. Five replicates were performed for each concentration, and the experiment was independently repeated three times.

### 2.4. Optimization of Protoplast Preparation

Protoplast preparation conditions were optimized primarily using a sequential one-factor-at-a-time approach, in which the selected condition from each assay was applied to subsequent optimization steps where applicable; digestion time was evaluated in parallel during the early optimization experiments.

#### 2.4.1. Hyphal Preparation

For mycelial preparation, *P. disseminata* ZXD579 was cultured on MEA plates at 28 °C for 5–7 days. Mycelial plugs approximately 2 mm in diameter were excised from the colony margin and transferred to 100 mL of PDB in 250-mL Erlenmeyer flasks. Cultures were incubated at 28 °C and 120 rpm. To determine the optimal mycelial age for protoplast isolation, mycelia cultured for 12, 24, 36, or 48 h were harvested for enzymatic digestion. The collected mycelia were filtered through sterile gauze, washed twice with sterile ddH_2_O, and washed once with the corresponding osmotic stabilizer.

#### 2.4.2. Lytic Enzyme Combinations

To determine the most effective enzyme combination for protoplast release, four different mixtures of cell wall-degrading enzymes were tested (Table 1). All enzyme solutions were prepared in an osmotic stabilizer consisting of 0.6 M KCl supplemented with 50 mM CaCl_2_. Mycelia cultured for 24 h were resuspended in 30 mL of each enzyme solution and incubated at 28 °C with gentle shaking at 100 rpm for 3 h.

#### 2.4.3. Osmotic Stabilizer, Digestion Time, and Temperature

Based on the optimal enzyme combination, the effects of osmotic stabilizer, digestion time, and temperature on protoplast release were further examined. Four osmotic stabilizers, 0.7 M NaCl, 0.6 M KCl, MN buffer (0.3 M MgSO_4_ + 0.3 M NaCl), and 0.6 M KCl + 50 mM CaCl_2_, were compared for their effects on protoplast yield. Digestion time was evaluated at 1, 2, 3, and 4 h, and digestion temperature was tested at 25, 28, 30, and 37 °C. Unless otherwise stated, all experiments were performed using 3 g fresh mycelia in 30 mL of enzyme solution.

#### 2.4.4. Protoplast Harvesting and Regeneration Assay

After enzymatic digestion, the digestion mixture was filtered through two layers of sterile Miracloth to remove undigested hyphal fragments. The filtrate was centrifuged at 5000 rpm for 10 min at 4 °C, and the protoplast pellet was gently washed twice with ice-cold STC buffer. Finally, the protoplasts were resuspended in STC buffer and counted using a haemocytometer. Protoplast morphology was observed using an Axio Imager A2 microscope (Carl Zeiss, Munich, Germany).

To assess the viability and regeneration capacity of the isolated protoplasts, 100 μL of protoplast suspension (1 × 10^7^ protoplasts/mL) was mixed with 7 mL of RM medium in a 50-mL centrifuge tube and incubated at 25 °C in a rotary shaker at 85 rpm for 16–18 h. Subsequently, the culture was diluted appropriately with STC buffer, spread onto RM solid medium, and incubated at 28 °C for 3–5 days. The regeneration rate was calculated as the percentage of protoplasts that formed visible colonies relative to the total number of protoplasts plated. As a control, protoplasts were diluted in sterile double-distilled water (instead of STC) and plated in the same manner to confirm that the regenerated colonies originated exclusively from protoplasts and not from residual hyphal fragments. The experiment was performed in triplicate.

### 2.5. PEG-Mediated Protoplast Transformation

PEG-mediated transformation of *P. disseminata* ZXD579 protoplasts was performed with modifications to previously described protocols [25,26,27].

For transformation, 750 μL of protoplast suspension (approximately 1 × 10^7^ protoplasts/mL) was gently mixed with 15 μg of pCT74 plasmid DNA in a 50-mL centrifuge tube. Next, 7.5 μL of heparin sodium solution (0.5 mg/mL) was added, followed by 1 mL of SPTC buffer. The mixture was gently inverted 2–3 times and incubated on ice in the dark for 30 min to allow DNA adsorption to the protoplast membrane. Subsequently, 800 μL of SPTC buffer was added slowly, and the mixture was incubated at room temperature in the dark for an additional 20 min to facilitate PEG-mediated DNA uptake.

After transformation, 25 mL of RM liquid medium was added to the protoplast–DNA mixture and incubated at 25 °C in a rotary shaker at 100 rpm for 16–18 h to allow protoplast regeneration. The regenerated protoplast suspension was then mixed with an equal volume of molten RM solid medium (cooled to approximately 50 °C) containing 130 μg/mL hygromycin B and poured into 90-mm Petri plates. After the medium solidified, the plates were overlaid with 20 mL of RM solid medium containing 260 μg/mL hygromycin B. The plates were incubated at 25 °C in the dark for 7–10 days. Hygromycin-resistant transformants emerging on the selection medium were directly purified by microscopic isolation of individual hyphal fragments from the selection plates. The isolated hyphal fragments were transferred to fresh PDA medium for further growth and subsequent molecular and phenotypic analyses.

Transformation efficiency was calculated as the number of hygromycin-resistant transformants obtained per microgram of plasmid DNA used. Control experiments with untransformed protoplasts were performed in parallel to confirm the adequacy of the selective pressure.

### 2.6. Molecular Confirmation of Transformants

Genomic DNA was extracted from fresh mycelium grown on MEA at 25 °C using the Ezup Column Fungi Genomic DNA Purification Kit (Sangon Biotech (Shanghai) Co., Ltd., Shanghai, China; Cat. No. B518259-0100) according to the manufacturer’s instructions and used as the template for PCR. Primer pairs targeting *eGFP* and *hph* are listed in Table 2. The PCR conditions were as follows: initial denaturation at 94 °C for 2 min; 32 cycles of denaturation at 94 °C for 30 s, annealing at 55 °C for 30 s, and extension at 72 °C for 30 s; and a final extension at 72 °C for 2 min. Plasmid pCT74 and genomic DNA from the wild-type strain served as the positive and negative controls, respectively.

PCR-positive transformants were further examined for GFP fluorescence using an Axio Imager A2 microscope (Carl Zeiss Microscopy GmbH, Jena, Germany) under 488-nm excitation. Hyphae and conidia of the wild-type strain were observed in parallel as negative controls.

To assess the stability of the transformed phenotype during serial subculture, 10 hygromycin-resistant candidate transformants were selected from the 15 isolates used for the initial PCR screening. The selected candidates included both PCR-positive and PCR-negative isolates based on the initial detection of *eGFP* and *hph*. These isolates were serially subcultured on non-selective MEA for five consecutive generations. Genomic DNA was extracted from the first-generation (G1) and fifth-generation (G5) cultures, and the presence of *eGFP* and *hph* was examined by PCR using the primer pairs and amplification conditions described above. The PCR profiles of corresponding G1 and G5 cultures were compared.

### 2.7. Phenotypic Characterization

To assess the effect of transformation on vegetative growth, the mycelial growth rates of the wild-type strain ZXD579 and three randomly selected positive transformants were compared. Mycelial plugs (9 mm in diameter) were taken from the actively growing edges of 3-day-old cultures and transferred to the center of fresh MEA plates. The plates were incubated at 28 °C in the dark. Colony diameters were measured daily for 7 days along two perpendicular axes passing through the center of each colony, and the mean of the two measurements was used as the colony diameter. Three biological replicates were maintained for each strain.

### 2.8. Preliminary Pathogenicity Assessment

A preliminary pathogenicity assessment was conducted on healthy *P. massoniana* seedlings to determine whether genetic transformation and GFP expression altered symptom development. A needle-wound inoculation method was used following the study of Li et al. [28], with modifications for qualitative comparison.

The wild-type strain ZXD579 and three randomly selected, PCR- and fluorescence-positive transformants were cultured in PDB at 28 °C for 2 days. Mycelial suspensions were prepared by homogenization and adjusted to a uniform concentration. For inoculation, a sterile needle was used to create a single, standardized wound on the surface of healthy pine needles, after which 20 μL of the respective mycelial suspension was applied to the wound. Three seedlings, each with 10 needles, were inoculated per strain. As a negative control, sterile distilled water was applied to wounded needles in the same manner.

All inoculated seedlings were maintained in a greenhouse at 28 °C with 90% relative humidity. Disease symptom development, including lesion initiation and expansion, was monitored and photographed daily for 15 days. The severity of disease symptoms was evaluated using a pre-defined scale based on the proportion of the needle length that became necrotic: − (no symptoms), + (lesion length < 1/3 of needle), ++ (lesion length between 1/3 and 2/3 of needle), and +++ (lesion length > 2/3 or entire needle necrotic). The consistency of symptom development between transformants and the wild-type strain was the primary criterion for comparison. At the end of the experiment, re-isolation of the inoculated fungus from the lesion margins was performed for all strains, and the recovered isolates were identified by morphological observation and ITS sequence analysis.

### 2.9. Statistical Analysis

All experiments included at least three replicates. Data are presented as means ± standard deviation (SD). Normality of residuals and homogeneity of variances were assessed using the Shapiro–Wilk test and Levene’s test, respectively. For comparisons among multiple groups (e.g., enzyme combinations, osmotic stabilizers, digestion times, and temperatures), one-way analysis of variance (ANOVA) followed by Tukey’s honest significant difference (HSD) post hoc test was used. A *p* < 0.05 was considered statistically significant. Statistical analyses were performed using SPSS version 26.0 (IBM Corp., Armonk, NY, USA).

## 3. Results

### 3.1. Establishment of a Hygromycin B Selection System

**Figure 1:**
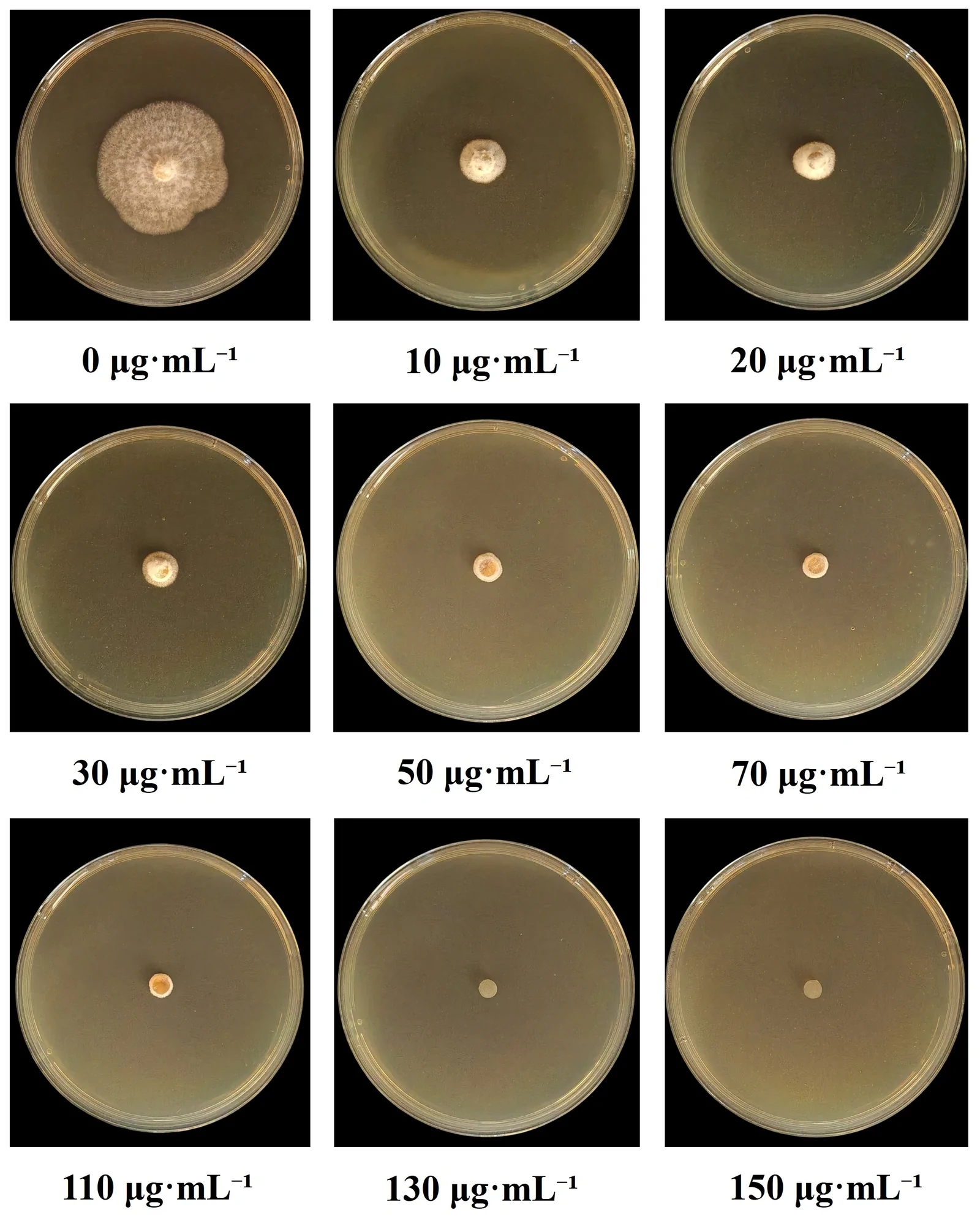
Sensitivity test of *P. disseminata* ZXD579 to hygromycin B. The wild-type strain was cultured on MEA plates supplemented with different concentrations of hygromycin B (0–150 μg/mL) at 28 °C for 5 d. Complete inhibition of mycelial growth was observed at 130 μg/mL.

To determine the appropriate selection concentration for hygromycin-resistant transformants, the growth of wild-type *P. disseminata* ZXD579 was examined on MEA supplemented with different concentrations of hygromycin B. After 5 days of incubation at 28 °C, the wild-type strain grew normally on plates without hygromycin B. In contrast, mycelial growth was progressively inhibited as the antibiotic concentrations increased. Complete inhibition was observed at 130 μg/mL, and at the concentration of 150 μg/mL, the mycelium remained non-growing (Figure 1). Therefore, 130 μg/mL hygromycin B was selected for transformant purification.

### 3.2. Optimization of Protoplast Preparation for P. disseminata

To establish a reproducible protoplast preparation protocol for *P. disseminata*, we systematically evaluated lytic enzyme composition, mycelial age, osmotic stabilizer, digestion time, and digestion temperature.

**Figure 2:**
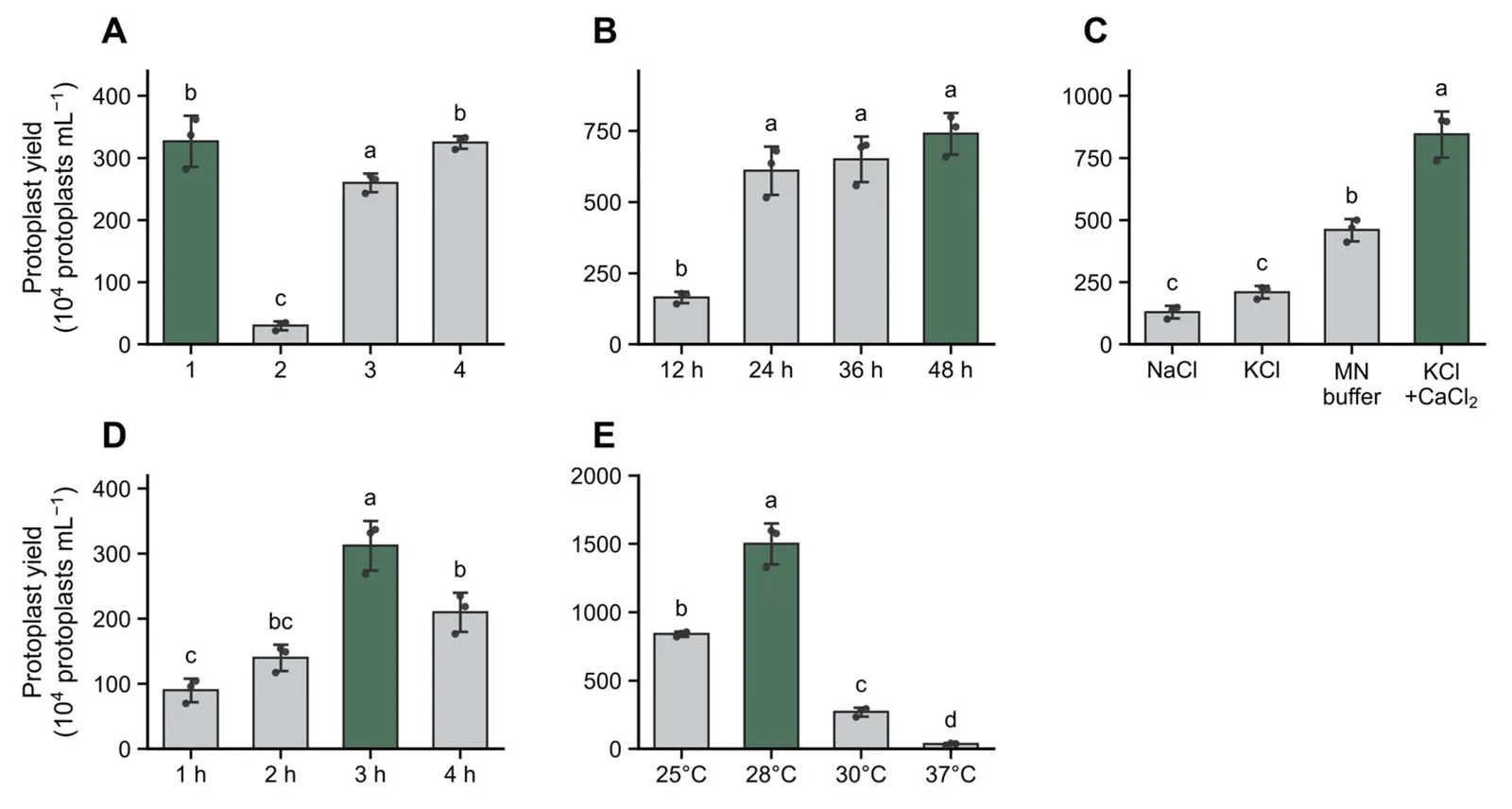
Effects of different factors on protoplast yield of *P. disseminata* ZXD579. (**A**) Enzyme combinations. (**B**) Mycelial age. (**C**) Osmotic stabilizers. (**D**) Digestion time. (**E**) Digestion temperature. Data are means ± SD of three replicates. Different letters indicate significant differences (*p* < 0.05).

Among the four enzyme combinations tested, combination 1, containing 1% lysing enzyme, 0.1% driselase, and 1% lysozyme, produced a high protoplast yield of (3.27 ± 0.41) × 10^6^ protoplasts/mL, comparable to that obtained with combination 4 (Figure 2A). The addition of cellulase did not further improve protoplast release, and increasing the driselase concentration from 0.1% to 0.5% also failed to increase yield.

Mycelial age markedly affected protoplast release. Among the tested culture times, mycelia cultured for 48 h yielded the highest number of protoplasts, reaching (7.4 ± 0.73) × 10^6^ protoplasts/mL (Figure 2B). Although the yield from mycelia cultured for 24 h was not significantly different from that of 36- or 48-h cultures (*p* > 0.05), the 24-h culture was selected for subsequent experiments because it required the shortest preparation time while maintaining a high protoplast yield.

The osmotic stabilizer significantly influenced protoplast recovery. Among the four stabilizers tested, the solution containing 0.6 M KCl and 50 mM CaCl_2_ produced the highest yield (8.45 ± 0.93 × 10^6^ protoplasts/mL), followed by MN buffer (0.3 M MgSO_4_ + 0.3 M NaCl), while 0.7 M NaCl and 0.6 M KCl alone gave significantly lower yields (Figure 2C).

In the time-course assay, protoplasts began to emerge after 1 h of digestion, and the yield increased with prolonged digestion. The protoplast yield reached a plateau at 3 h, with (3.125 ± 0.38) × 10^6^ protoplasts/mL under the tested conditions (Figure 2D).

Digestion temperature had a strong effect on protoplast production. The highest yield was obtained at 28 °C, reaching (1.5 ± 0.15) × 10^7^ protoplasts/mL (Figure 2E). When the temperature increased to 30 °C, the protoplast yield decreased sharply to approximately (0.27 ± 0.032) × 10^7^ protoplasts/mL, and further reduction in protoplast production was observed at 37 °C.

Collectively, the optimized protoplast preparation conditions were mycelia cultured for 24 h, digested for 3 h at 28 °C with 1% lysing enzyme, 0.1% driselase, and 1% lysozyme in 0.6 M KCl supplemented with 50 mM CaCl_2_. Under these conditions, the protoplast yield reached (1.51 ± 0.15) × 10^7^ protoplasts/mL.

### 3.3. Protoplast Morphology and Regeneration

**Figure 3:**
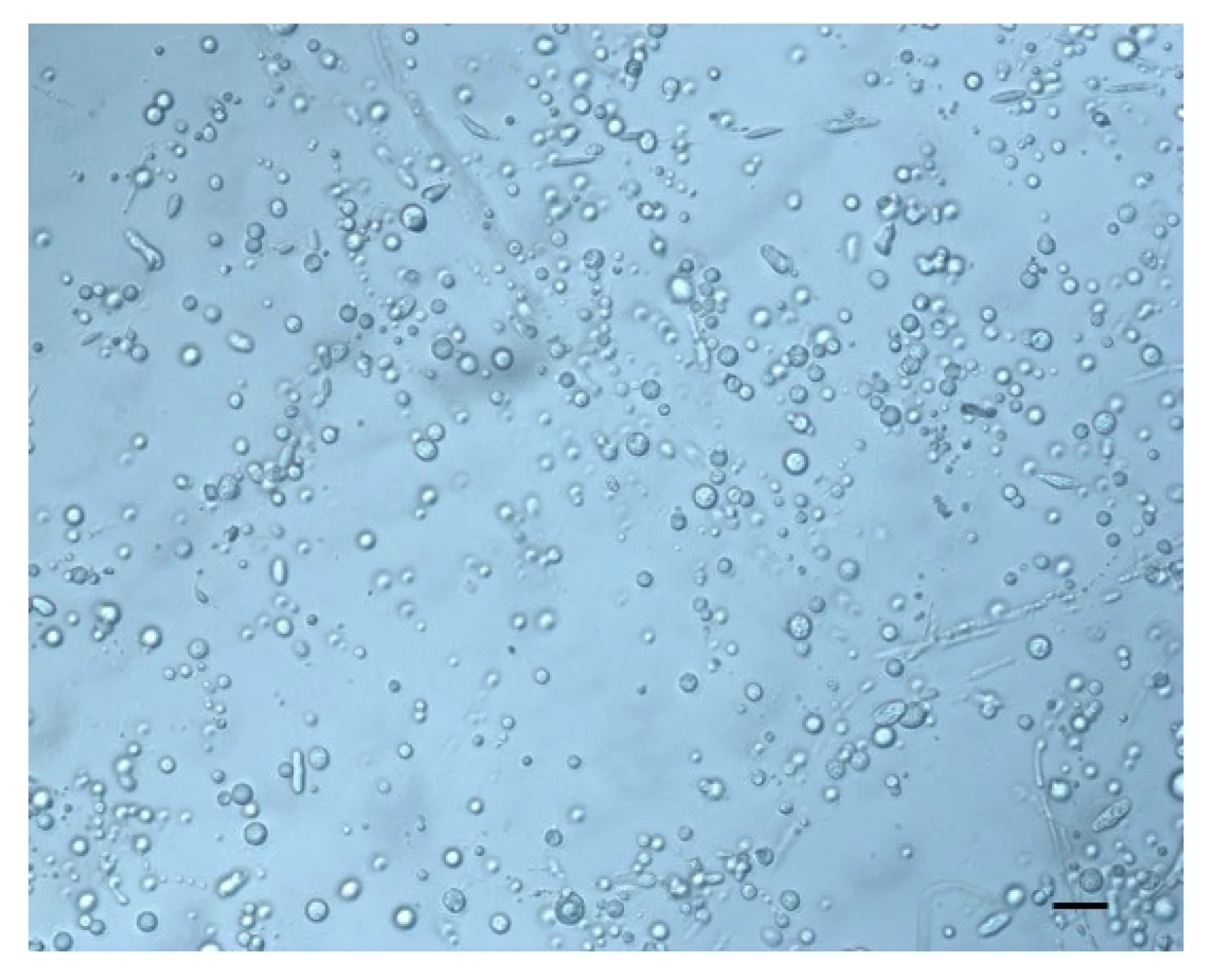
Microscopic observation of protoplasts isolated from *P. disseminata* ZXD579. Protoplasts were spherical in shape with diameters ranging from 7 to 15 μm (average approximately 9 μm). Bar = 30 μm.

**Figure 4:**
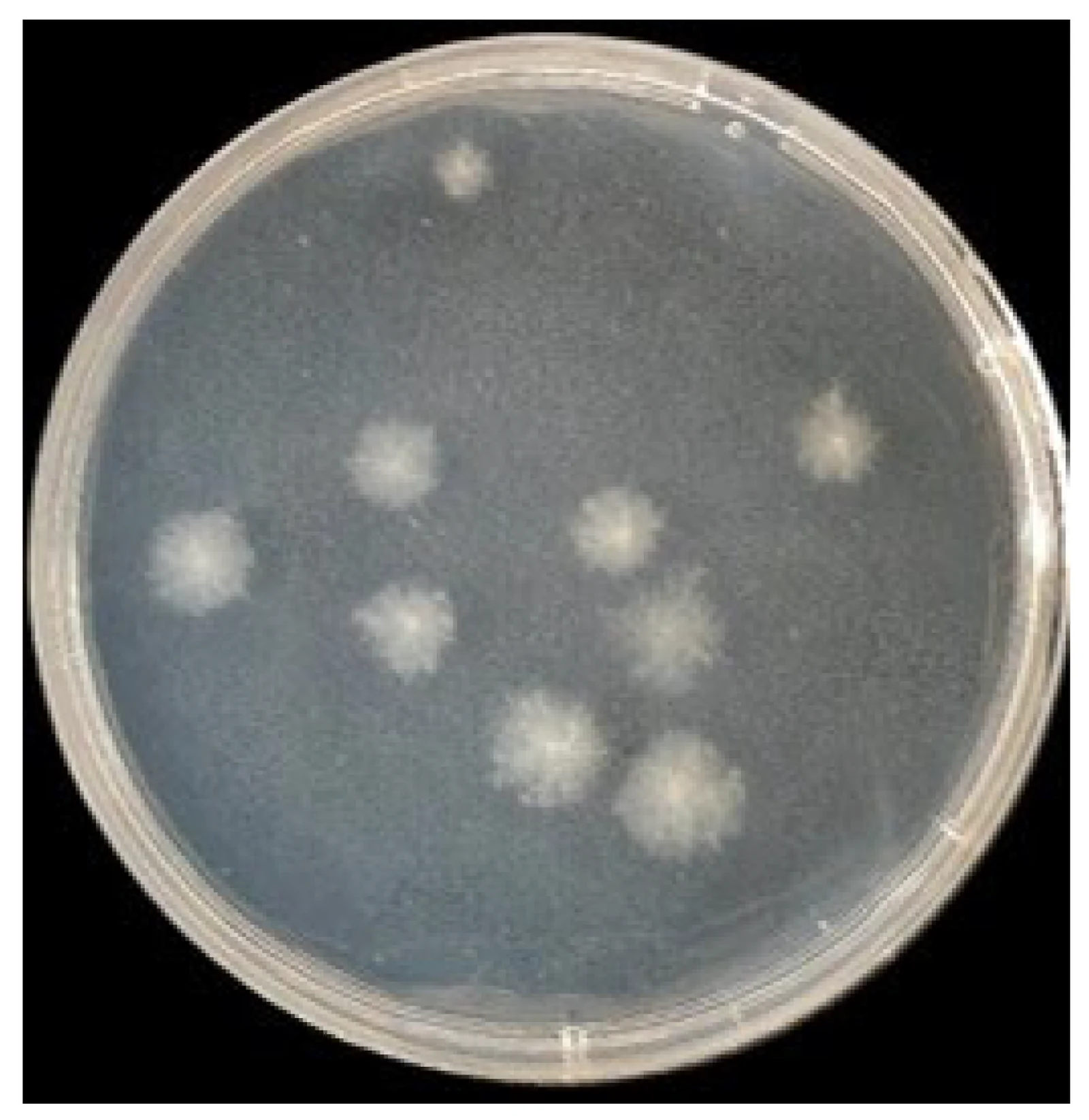
Growth of *P. disseminata* ZXD579 transformants on hygromycin B-containing selective medium. Putative transformants emerged on RM agar medium containing 130 μg/mL hygromycin B after 5–7 d of incubation at 25 °C.

Under the optimized conditions, the released protoplasts were spherical and uniform, with diameters of 7–15 μm (Figure 3). After transfer to regeneration medium, protoplasts regenerated and formed visible colonies within 3–5 days (Figure 4). The regeneration rate exceeded 90%, indicating that the isolated protoplasts remained viable and were suitable for PEG-mediated transformation. No colonies formed in the osmotic-lysis control, confirming that the regenerated colonies originated from protoplasts rather than residual hyphal fragments.

### 3.4. PEG-Mediated Transformation and Molecular Confirmation

Following PEG-mediated transformation with pCT74, hygromycin-resistant transformants emerged on selective RM medium after 5–7 days. No colonies were observed in the untransformed protoplast control, indicating that the hygromycin concentration effectively suppressed false-positive growth. To obtain purified transformants and reduce potential heterokaryotic effects, hygromycin-resistant transformants were subjected to microscopic isolation of individual hyphal fragments from the selection plates before PCR verification. The isolated hyphal fragments were transferred to fresh PDA medium for further growth and subsequent molecular and phenotypic analyses (Figure 4). Transformation efficiency was 4.2 ± 0.6 transformants per μg plasmid DNA.

**Figure 5:**
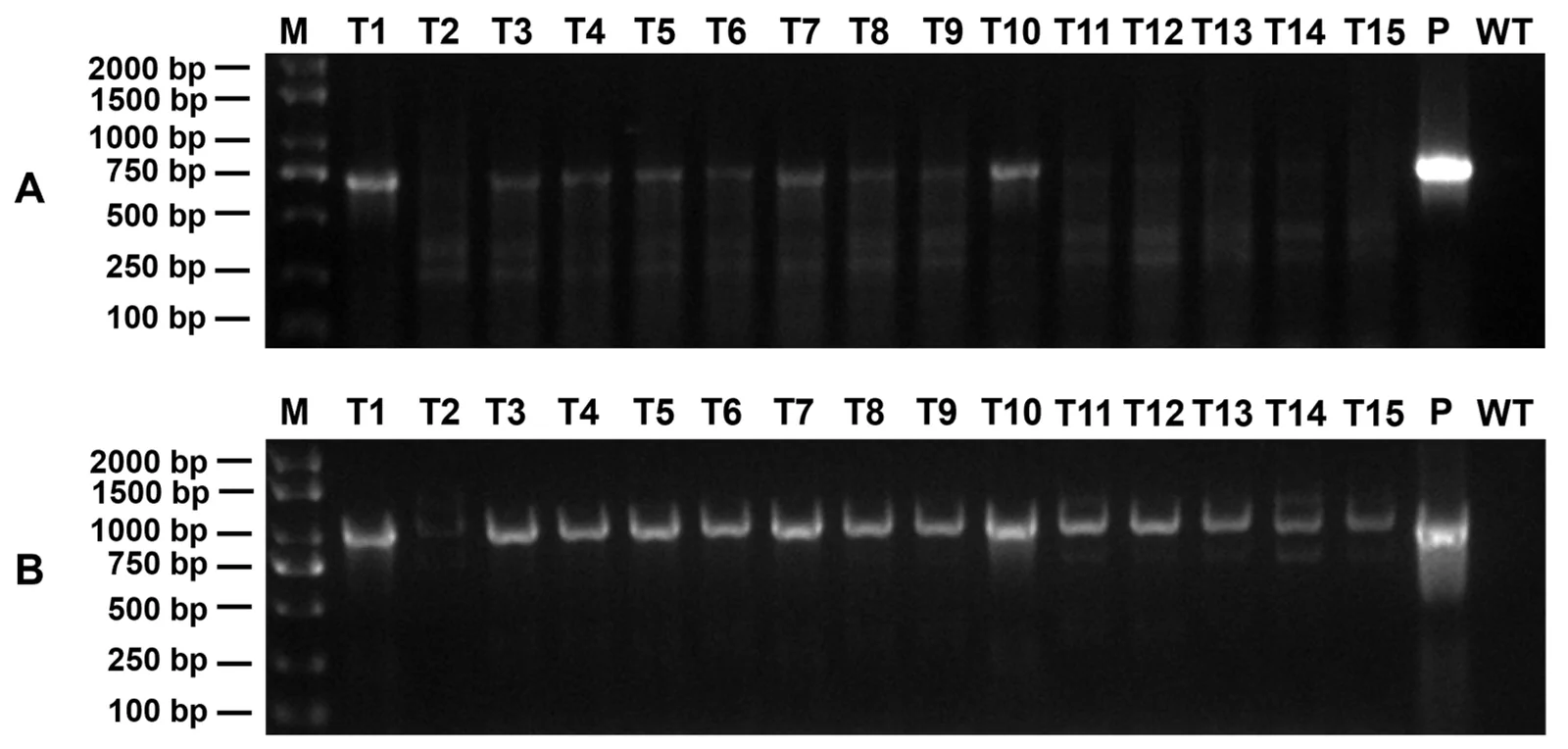
PCR detection of *eGFP* and *hph* marker sequences in *P. disseminata* candidate transformants. (**A**) Amplification of *eGFP* fragment (720 bp). (**B**) Amplification of *hph* fragment (1026 bp). M: 2000 bp DNA marker; Lanes 1–15: genomic DNA from randomly selected transformants; P: plasmid pCT74 (positive control); WT: wild-type strain (negative control).

To verify the presence of the reporter and selectable marker genes, 15 hygromycin-resistant transformants were randomly selected for PCR analysis. Nine transformants yielded the expected 720-bp *eGFP* and 1026-bp *hph* amplicons, consistent with the positive plasmid control, whereas no amplification was detected in the wild-type strain (Figure 5A,B). These results indicated the presence of the pCT74-derived marker sequences in *P. disseminata*, and the PCR-positive rate among the randomly selected hygromycin-resistant transformants was 60%.

### 3.5. GFP Labeling

**Figure 6:**
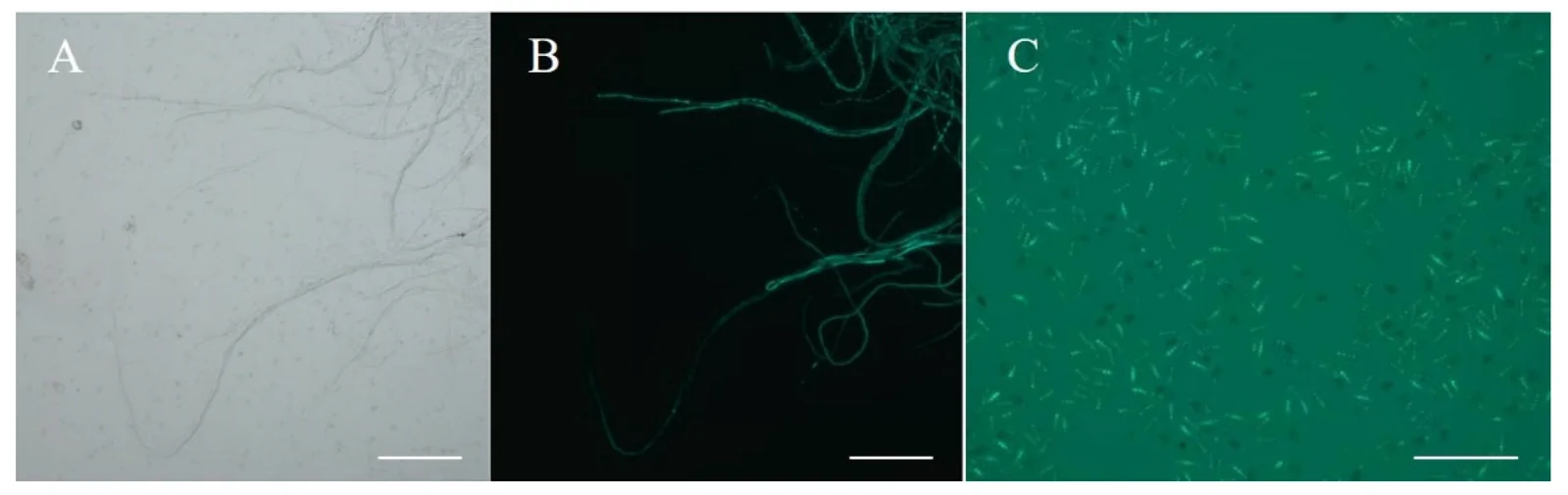
Fluorescence detection of *eGFP* expression in hyphae and conidia of *P. disseminata* transformants. (**A**) Hyphae of transformant under brightfield. Bar = 100 μm. (**B**) Hyphae of a transformant under 488-nm excitation showing green fluorescence. Bar = 100 μm. (**C**) Conidia of a transformant under 488-nm excitation. Bar = 20 μm.

GFP fluorescence was examined in PCR-positive transformants. Under 488-nm excitation, all tested PCR-positive transformants exhibited strong green fluorescence throughout the hyphal cytoplasm (Figure 6A,B), and clear fluorescence was also detected in conidia (Figure 6C). Neither hyphae nor conidia of the wild-type strain showed detectable fluorescence under the same conditions, indicating functional expression of *eGFP* in *P. disseminata* transformants.

### 3.6. Stability of Transformants During Serial Subculture

**Figure 7:**
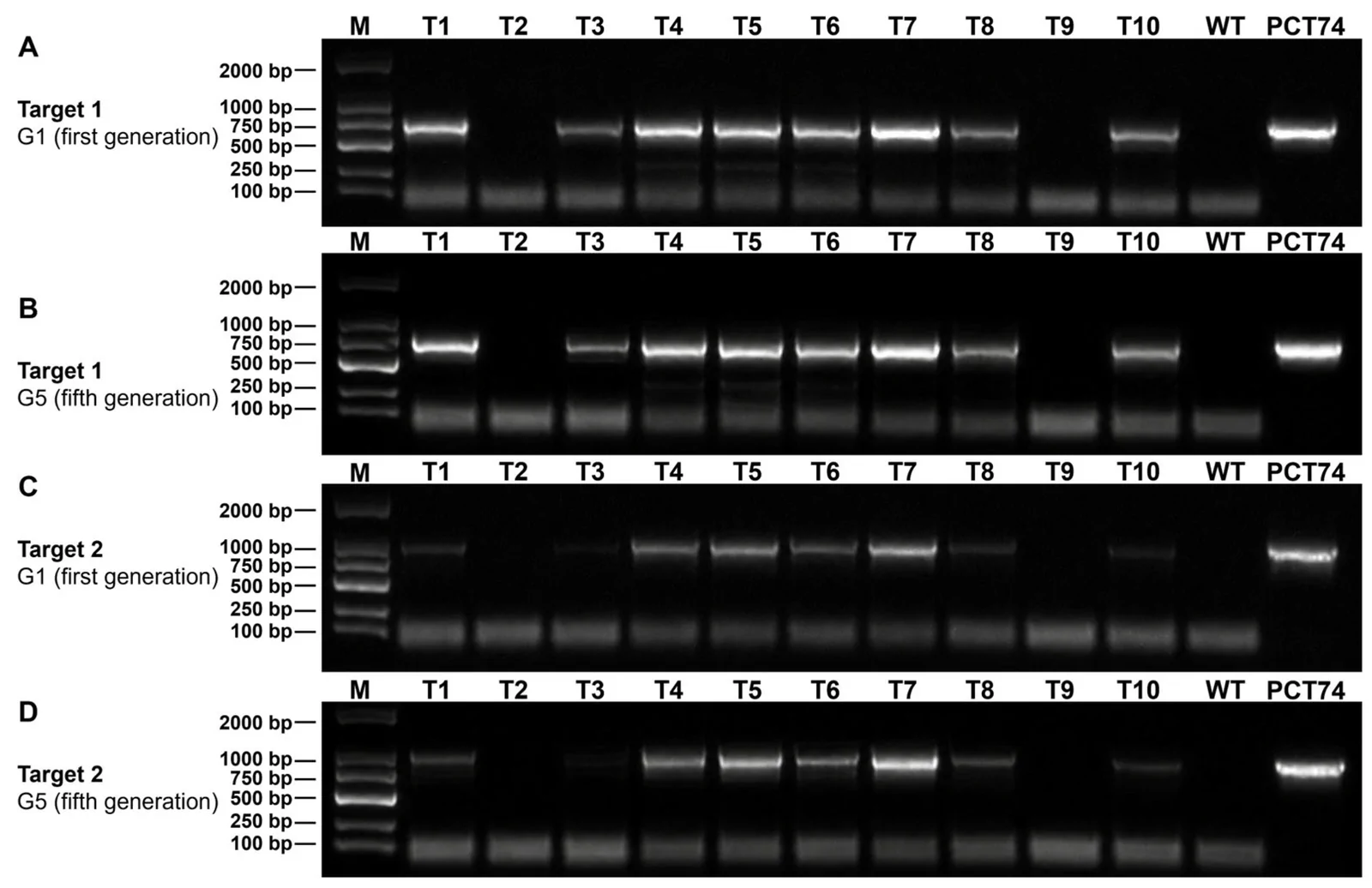
PCR analysis of *eGFP* and *hph* marker sequences in first- and fifth-generation cultures of *P. disseminata* candidate transformants. (**A**) Detection of the *eGFP* fragment (720 bp) in first-generation (G1) cultures. (**B**) Detection of *eGFP* in the corresponding fifth-generation (G5) cultures after five consecutive generations of non-selective subculture. (**C**) Detection of the *hph* fragment (1026 bp) in G1 cultures. (**D**) Detection of *hph* in the corresponding G5 cultures. M, DNA marker; T1–T10, hygromycin-resistant candidate transformants selected from the initial screening set; WT, wild-type strain; PCT74, pCT74 plasmid positive control.

To further evaluate the stability of candidate transformants during serial subculture, 10 hygromycin-resistant candidates selected from the initial screening set were analyzed by PCR at the first (G1) and fifth (G5) generations. These isolates included both PCR-positive and PCR-negative candidates identified during the initial screening. Candidates showing the expected *eGFP* (720 bp) and *hph* (1026 bp) amplicons in G1 generally retained the corresponding PCR profiles in G5, whereas candidates lacking detectable target amplification in G1 remained negative after serial subculture. No target amplification was detected in the wild-type control (Figure 7). These results indicate that the marker profiles of the selected candidate transformants were largely maintained during five consecutive generations of non-selective subculture.

### 3.7. Phenotypic Evaluation of GFP-Labeled Transformants

**Figure 8:**
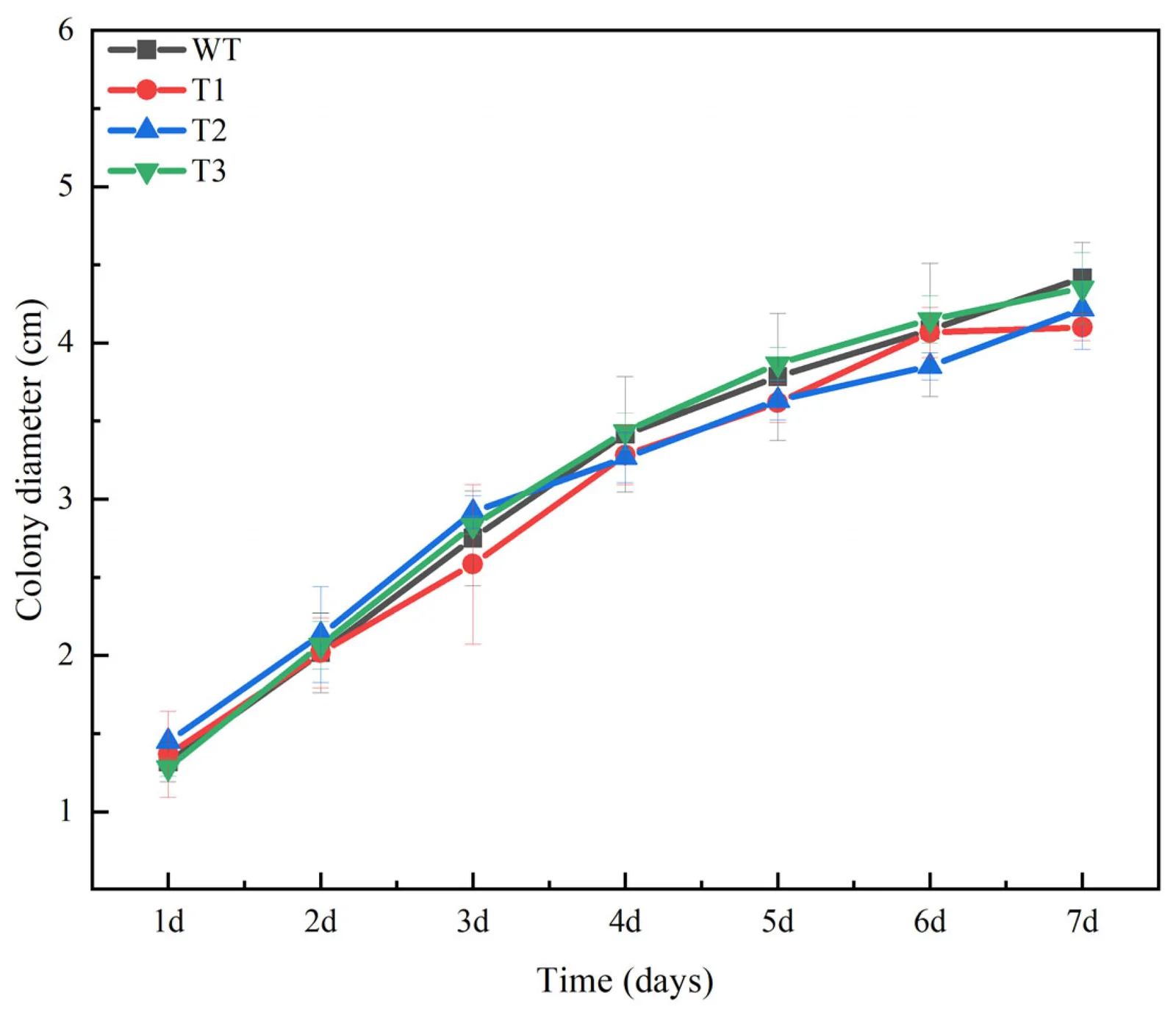
Mycelial growth of wild-type (WT) and three *eGFP*-tagged transformant strains (T1, T2, T3) of *P. disseminata* ZXD579 on MEA plates. Colony diameters were measured over 7 d. Values are means ± SD (*n* = 3). No significant differences were observed between the WT and transformants at any time point (*p* > 0.05).

Three independent GFP-positive transformants were grown on MEA to evaluate whether transformation affected vegetative growth. The transformants showed colony morphology similar to that of the wild-type strain, and radial growth rates did not differ significantly among strains (*p* > 0.05; Figure 8).

### 3.8. Pathogenicity Results

**Figure 9:**
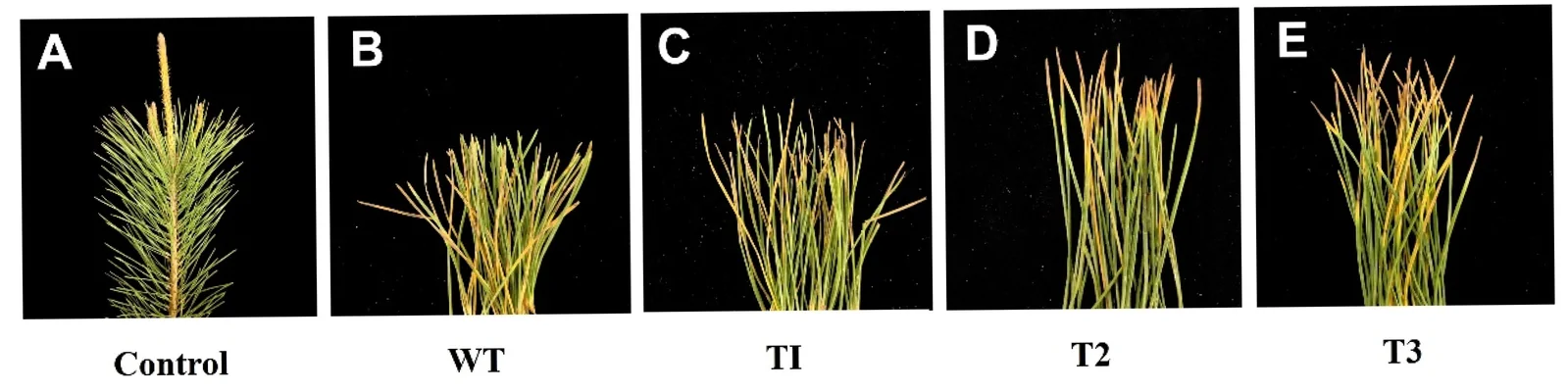
Preliminary pathogenicity assessment of the wild-type (WT) and GFP-labeled transformants of *P. disseminata* ZXD579 on wounded *Pinus massoniana* needles at 15 days post-inoculation (dpi). (**A**) Mock-inoculated control (sterile distilled water). (**B**) Wild-type strain (WT). (**C**–**E**) Three independent GFP-positive transformants (T1, T2, and T3). All transformants induced needle blight symptoms comparable to those of the WT strain, with brownish lesions restricted to the wound vicinity (lesion length < 1/3 of the needle, corresponding to a + rating), whereas mock-inoculated needles remained asymptomatic.

In the preliminary pathogenicity assessment, three GFP-positive transformants (T1, T2, and T3) and the wild-type strain (WT) induced needle blight symptoms on wounded *Pinus massoniana* needles, whereas mock-inoculated controls remained asymptomatic throughout the 15-day observation period (Figure 9). Symptoms developed slowly under the tested conditions. By 15 dpi, lesions in both WT- and transformant-inoculated needles remained restricted to the wound vicinity and extended less than one-third of the total needle length, corresponding to a + rating on the predefined severity scale. No visible differences were observed between transformants and the wild-type strain in lesion initiation, expansion pattern, or final lesion size; all strains produced comparably weak symptoms. At the end of the experiment, the fungi were re-isolated from lesion margins and identified as *P. disseminata* based on morphological characteristics and ITS sequence analysis.

## 4. Discussion

The fungus *Pestalotiopsis disseminata* exhibits diverse ecological associations, occurring in both endophytic and pathogenic contexts across different plant hosts [2,8,9]. Here, we established a PEG-mediated transformation and GFP-labeling system for *P. disseminata* based on optimized protoplast preparation and regeneration. The selected candidate transformants showed largely stable marker profiles during non-selective subculture, while PCR-positive transformants exhibited GFP expression in both hyphae and conidia and showed vegetative growth similar to that of the wild-type strain. To ensure reliable selection of transformants, we first determined the appropriate hygromycin B concentration, as antibiotic concentration is critical for transformant screening [15,29]. In this study, wild-type *P. disseminata* ZXD579 strain was completely inhibited at 130 μg/mL hygromycin B, a concentration higher than that reported for *Ceratocystis paradoxa* (40 μg/mL; [16]) and *Sclerotiophoma versabilis* [22], indicating species-specific tolerance differences. This concentration effectively suppressed false-positive growth while allowing recovery of candidate transformants.

The yield and quality of protoplasts are decisive factors for PEG-mediated transformation success, and species-specific optimization is necessary due to variations in cell wall composition and physiological status across fungi [15,20,22]. The cultivation period of donor mycelia is a critical factor affecting protoplast preparation efficiency. Although mycelia cultured for 24–48 h showed comparable protoplast yields, younger cultures are generally preferred for transformation procedures because they provide actively growing hyphae and reduce the time required for preparation. Therefore, mycelia cultured for 24 h were selected as a practical condition for obtaining suitable protoplasts with high transformation potential [30]. Among the enzyme combinations tested, the mixture of 1% lysing enzyme, 0.1% driselase, and 1% lysozyme in a stabilization solution containing 0.6 M KCl supplemented with 50 mM CaCl_2_ produced the highest protoplast yield, reaching (1.51 ± 0.15) × 10^7^ protoplasts/mL. This suggests that efficient cell-wall degradation required a balanced combination of enzymatic activities. The finding supports the view that mixed enzyme systems are more effective than single enzymes for degrading the complex fungal cell wall [31]. Among the osmotic stabilizers tested, the combination of 0.6 M KCl with 50 mM CaCl_2_ gave the highest protoplast yield, significantly outperforming 0.7 M NaCl, 0.6 M KCl alone, and MN buffer (0.3 M MgSO_4_ + 0.3 M NaCl). This result suggests that both osmotic protection and Ca^2+^-mediated membrane stabilization contribute to protoplast integrity [32]. The optimal digestion conditions of 28 °C for 3 h were also identified; temperatures above 30 °C sharply reduced yields, indicating that *P. disseminata* is particularly sensitive to thermal stress during enzymatic digestion. Under these optimized conditions, the regeneration rate exceeded 90%, indicating high protoplast viability suitable for transformation. However, the primarily one-factor-at-a-time optimization used in this study did not systematically evaluate potential interactions among the tested factors, which should be considered when interpreting the optimized conditions.

The PEG-mediated genetic transformation system yielded 4.2 ± 0.6 transformants per μg plasmid DNA, with a PCR-positive rate of 60% (9 out of 15 hygromycin-resistant transformants). The PCR-negative hygromycin-resistant candidates may represent false-positive resistant colonies or may reflect failure to detect the target sequences by PCR. The exact reason for this discrepancy was not determined in the present study, highlighting the importance of molecular verification of hygromycin-resistant candidates. This transformation efficiency was comparable to that reported for several filamentous fungi, including *Ceratocystis paradoxa* (4–5 transformants/μg DNA) [18]. Although transformation efficiency varies considerably among fungal species due to differences in cell wall composition, physiological characteristics, and transformation protocols [25,26,27], the system established here was sufficient for generating GFP-labeled transformants for downstream phenotypic and functional studies. Detection of both *eGFP* and *hph* amplicons indicated the presence of the vector-derived marker sequences.

Strong GFP fluorescence in hyphae and conidia of PCR-positive transformants indicated that the ToxA promoter in pCT74 effectively drove reporter expression in *P. disseminata*, consistent with reports in other ascomycetes [33]. The microscopic isolation of individual hyphal fragments from hygromycin-selection plates further improved transformant purification and reduced the potential influence of heterokaryosis, which is commonly encountered in filamentous fungi. Comparison of G1 and G5 cultures showed that the *eGFP* and *hph* PCR profiles of the selected candidates were largely maintained during five consecutive generations of non-selective subculture. Notably, both PCR-positive and PCR-negative candidates were included in this analysis, and their marker profiles generally remained consistent during serial subculture.

The three GFP-labeled transformants tested exhibited vegetative growth rates similar to that of the wild-type strain, consistent with findings in *C. paradoxa* and *S. versabilis* [18,22]. In the preliminary pathogenicity assessment, the wild-type strain and transformants produced comparably weak needle blight symptoms on *Pinus massoniana* at 15 days post-inoculation, with lesions restricted to the wound vicinity (<1/3 of needle length). The relatively weak symptom development may have been influenced by the inoculation conditions, host physiological status, or the duration of the observation period, although the contribution of these factors was not specifically evaluated in the present study. Although this qualitative assay was not designed to establish or quantify pathogenicity, similar symptom profiles suggest that transformation and GFP expression did not produce an obvious phenotypic defect under the tested conditions.

The current system is suitable for GFP labeling and phenotypic characterization. However, persistence of the marker sequences during serial subculture, as assessed by PCR, does not by itself demonstrate stable genomic integration. Southern blotting or genome-based analyses were not performed in the present study; therefore, the transgene copy number and insertion sites remain undetermined. The transformation system was also established and evaluated using only one *P. disseminata* strain (ZXD579), and its applicability to other strains remains to be evaluated. Nevertheless, this PEG-mediated genetic transformation system represents, to our knowledge, the first genetic manipulation platform for *P. disseminata* and provides a foundation for studies of fungal development, pathogenicity-related genes, fungus–host interactions, and secondary metabolite biosynthesis. Future studies could further extend this platform to targeted gene manipulation for functional characterization of genes in *P. disseminata*.

## Figures and Tables

**Table 1:** Enzyme combinations tested for protoplast isolation from *Pestalotiopsis disseminata* ZXD579.

Enzyme System	1	2	3	4
Lysing enzyme	1%	0	0	0.5%
Driselase	0.1%	0.1%	0.5%	0.1%
Lysozyme	1%	1%	1%	1%
Cellulase	0	1%	1%	0

0, not added.

**Table 2:** Primers used for PCR verification of transformants.

Primer Name	Primer Sequence (5′-3′)	Target Fragment Length (bp)
*eGFP*-F2	TTACTTGTACAGCTCGTCCATGCCG	720 bp
*eGFP*-R2	ATGGTGAGCAAGGGCGAGGAGCTG	
*eGFP*-H-F2	ATGAAAAAGCCTGAACTCACCGCG	1026 bp
*eGFP*-H-R2	TATTCCTTTGCCCTCGGACGAGTG	

## Data Availability

The data presented in this study are available from the corresponding author upon reasonable request. Sequence accession numbers are provided in the manuscript.
